# Report of Diseases in the Public and Private Practice of One of the Physicians of the Finsbury Dispensary, from the 20th of November to the 20th of December

**Published:** 1806-01

**Authors:** J. Reid

**Affiliations:** Grenville Street, Brunswick Square


*Report of Diseases in the public and private Practice of one of the Physicians of the Finsbury Dispensary, from the 20th of November to the 20th of December.*

| | | | |
|---|---|---|---|
| Catarrhus | 23 | Hysteria | 3 |
| Diarrhœa et Dysenteria | 11 | Hypochondriasis | 8 |
| Pneumonia | 1 | Amenorrhœa et Chlorosis | 9 |
| Tussis et Dyspepsia | 8 | Menorrhagia | 6 |
| Phthisis | 5 | Asthenia | 19 |
| Rheumatismus | 7 | Ophthalmia Syphilitica | 1 |
| Hydrothorax | 2 | Ophthalmia Scrophulosa | 1 |
| Asthma | 16 | Hydrocephalus | 1 |
| Anasarca | 4 | Tabes Mesenterica | 9 |
| Pneumatosis | 1 | Morbi cutanei | 17 |

Fashion,

Fashion, that destroying angel, has scarcely commenced her career of depredation amongst the dissipated inhabitants of this metropolis. This is so far fortunate, as during the rigors of mid-winter the habits and amusements of the higher classes, and of those who are ambitious of imitating them, would prove more especially injurious, and more extensively fatal in their operation.

In the fashionable world the harvest of disease is not as yet fully ripe; but the inferior and intermediate ranks still continue in this, as in the preceding month, to exhibit a more than ordinary profusion of catarrhal and other kindred affections.

To individuals of every order in the community it appears, at this season of the year particularly, suitable and important to suggest the expediency of avoiding the sudden application of the stimulus of artificial warmth, after the excitability has unduly accumulated in consequence of its temporary deprivation.

What is called catching a *cold*, ought to be called catching a *heat*; it is produced not by going out of a heated apartment into a frigid atmosphere, but out of the latter into the former. The best way, indeed, of guarding against the danger of a chilling medium is, immediately before exposure to its influence, to charge the body with a superabundant quantity of caloric.

The experiments and reasonings of Fordyce, Darwin, Currie,* Beddoes, and still more recently of Dr. Stock of Bristol, have co-operated to confirm, and fully establish this doctrine. But it was first suggested by the originality of that man, the impetus of whose powerful and ponderous mind turned at once into a new channel all medical practice and speculation.† Even Brown, however, had only the merit of laying the first stone of a still unfinished edifice. He drew a rude and inaccurate outline, which has since, by other hands, to a certain degree, been corrected and

* To mention the name of Currie, is scarcely possible, without expressing a deep regret that the name *alone* remains of one who possessed all the brilliancy and all the ardour of genius; who, with professional acquisitions and talents the most eminent and practical, united an elegance of taste and a degree of classical erudition, which made him, if not quite, nearly the finest writer of his age.

† "The philosophy of Brown, which is the *philosophy of organized nature*, was produced in Scotland, and has been cultivated and improved in Germany. It is despised in France, where it is still *imperfectly known*."
Villers on the Reformation.

and filled up. But for probably a long succession of future intellects is it left to accomplish and complete the moral and physical philosophy of the human frame.

J. REID.

*Grenville Street, Brunswick Square.*

